# Dialkyl phosphate metabolites of organophosphorus in applicators of agricultural pesticides in Majes – Arequipa (Peru)

**DOI:** 10.1186/1745-6673-1-27

**Published:** 2006-12-19

**Authors:** Sandra Yucra, Kyle Steenland, Arturo Chung, Fredy Choque, Gustavo F Gonzales

**Affiliations:** 1 Instituto de Investigaciones de la Altura, Faculty of Sciences and Philosophy, Universidad Peruana Cayetano Heredia, Lima, Peru; 2 Department of Biological and Physiological Sciences (Laboratory of Investigation and Development), Faculty of Sciences and Philosophy, Universidad Peruana Cayetano Heredia, Lima, Peru; 3 Rollins School of Public Health, Emory University, Atlanta, Georgia USA; 4 Universidad Nacional de San Agustin, Arequipa, Peru

## Abstract

**Background:**

Organophosphorus (OPs) pesticides are the most commonly used pesticides in Peruvian agriculture. The population at risk for OPs exposure includes formulators, applicators and farmers. Majes Valley is the most important agricultural center of the Southern region of Peru. The present study was aimed to determine the knowledge about using OPs, safety practice and urinary dialkylphosphate metabolites on OP applicators in the Majes Valley, Peru.

**Methods:**

This study was based on a questionnaire which included socio-demographic characteristics, knowledge of safety practices to handling OPs, characteristics of pesticide application and use of protective measures to avoid pesticide contamination. Exposure was assessed by measuring six urinary OP metabolites (DMP, DMTP, DMDTP, DEP, DETP, and DEDTP) by gas chromatography using a single flame photometric detector. The sample consisted of 31 men and 2 women aged 20 – 65 years old.

**Results:**

76% of applicators had at least one urinary dialkylphosphate metabolite above the limit of detection. The geometric mean (GM) and the geometric standard deviation (GSD) of DMP and DEP were 5.73 ug/g cr. (GSD 2.51), and 6.08 ug/g cr. (GSD 3.63), respectively. The percentage of applicators with detectable DMP, DMDTP, and DMTP in urine was 72.72%, 3.03%, and 15.15%, respectively, while the corresponding figures for DEP, DETP, and DEDTP were 48.48%, 36.36% and 15.15%, respectively. There was no significant association between the use of protection practices and the absence of urine OPs metabolites suggesting inadequate protection practices.

**Conclusion:**

The pesticide applicators in Majes Valley have significant exposure to OP pesticides, probably due to inappropriate protective practices. Future work should evaluate possible health effects.

## Background

Organophosphorus (OPs) are broadly used in pest control in agriculture [1]. Pesticide exposure can occur through a number of sources such as contaminated soil, dusty work clothing, water, contaminated food, and drift of a pesticide off target deposition [2,3]. A high risk of occupational human exposure to OPs may occur in pesticide applicators if they do not practice adequate protective measures [4].

The measurement of blood cholinesterase is used as a biological marker of OPs contamination. This is based on the fact that organophosphate pesticides inhibit the activity of both the cholinesterase (ChE) enzymes in the red blood cells (RBC Che) and in the serum ChE (AchE) [5]. A 50% reduction in serum ChE activity from the baseline is an indicator of acute organophosphate toxicity. The RBC ChE activity, which is less rapidly depressed than the serum ChE activity (AChE), is a measure of more chronic exposure to organophosphates [5]. Although cholinesterase monitoring has the advantage of providing a measure of physiological response, it has disadvantages as well [6]. Interpretation of AChE monitoring is complicated by inter- and intra-individual variation in enzymatic activity and use of other cholinesterase-inhibiting pesticides as carbamates [6]. Likewise, the absence of baseline values for an individual subject makes of difficult to know if an observed level of AChE or RBC ChE activity represents a depression by exposure to an OP or if the value is normal for the subject [7].

An alternative approach to biological monitoring for OPs is based on the analysis of six dialkylphosphates metabolites in urine as DMP (Dimethylphosphate), DMTP (Dimethylthiophosphate), DMDTP (Dimethyldithiophosphate), DEP (Diethylphosphate), DETP (Diethylthiophosphate), and DEDTP (Diethyldithiophosphate) [6,8-10]. The determination of these metabolites is used to monitor occupational exposure to OP pesticide [11], OP metabolites are often the preferred method for pesticide measurements because their collection is non invasive and they are easily measured [12], and because they are more sensitive than ChE activity (can de detected at lower levels of OP exposure. [8]. First morning void samples may accurately represent total daily exposure [13]. However, there are also disadvantages. For instance, urine output varies, and therefore the concentration of OPs may vary. This may solved by creatinine correction in urine samples. Metabolites measured in urine are also not pesticide specific, and they may enter the body from other exposure sources [6]. Despite these disadvantages, measurements of dialkylphosphates metabolites are one of the commonly used markers of OPs exposure.

The OPs are hydrolyzed rapidly to six dialkylphosphate metabolites detectable in the urine, which may be measured for several days after exposure [7]. While there are many studies reported in the literature of measurements of dialkylphosphate metabolites in urine of agriculture workers, to our knowledge there are no reports countries in South America.

One of the main agriculture centers in Peru is located in the Majes Valley located at the Southern part of the country, in the department of Arequipa. The present study has been designed to determine socio-demographic characteristics and safety practices of OP pesticide applicators in the Majes Valley and to determinate exposure to OPs through the presence of six organophosphates metabolites in urine samples from these workers.

## Methods

### Study design

This is a cross-sectional descriptive study, based on interviews and collection of urine samples of 33 OP pesticides applicators (31 men and 2 women). The requirements to participate in the study were to have worked with pesticides and lived in Majes at least for two years before the study. Age of subjects ranged from 20 to 65 years.

The study was approved by the Institutional Review Board (IRB) at the Universidad Peruana Cayetano Heredia in Lima, Peru. A signed informed consent was obtained from each study participant following procedures established by the IRB at the Universidad Peruana Cayetano Heredia, Lima, Peru and at the Emory University, School of Medicine, Atlanta-Georgia-USA.

### Study area

Majes is an agricultural area located in Caylloma, Arequipa. It is one of the main areas of agricultural production in the Southern part of Peru. It is situated at 1420 m. above sea level. The temperate climate makes agricultural production possible almost all the year. OP pesticides are used on a variety of crops including potatoes, alfalfa, onions, tomatoes, garlic, apples and grapes. The three first are associated with OPs pesticide applications, especially potatoes., which are applied during two different seasons each year. Pesticide applicators are exposed to OPs during prolonged periods of time. Methylated pesticides such methamidophos are the most frequently used OP pesticide in the Majes valley.

### Population recruitment

The applicators participating in the study were identified and recruited by agronomic engineers working in Majes Valley. From the universe of applicators in Majes, 59 of them accepted to participate in the study. From these, only 33 satisfied the inclusion criteria. The inclusion criteria were: i) To be working as pesticide applicator for at least 2 years; ii) To have used pesticide the last week before questionnaire application; iii) To have used pesticide the day before the urine collection; iv) To agree to participate in the study.

Before the application of the questionnaire to the participants, we conducted a pilot study with 5 pesticide applicators to learn if they understood the questions, and then modified the questionnaire accordingly.

All participants in the study were instructed to carry out work activities according to their normal practice. The questionnaire was administered by trained interviewers to each pesticide applicator to obtain information on sociodemographic characteristics; agricultural work practice, and knowledge and practice of safety guidelines for pesticide use.

Applicators were asked to define how frequently theyused OPs pesticides. Data related to the kind of pesticides used, kind of protective measures used during application, and management of pesticides and clothes after pesticide application were also recorded.

### Urine collection, storage

One day after a OP pesticide application, each worker was provided with one polyethylene urine collection bottle and instructed to collect an urine sample from the first morning void. All the collected urine samples were immediately placed inside a plastic container with ice and transported to the medical center for freezing at -20°C. The time between urine collections to freezing processing was 10–15 minutes. After collection was completed, all samples were shipped frozen to the Pacific Toxicology Lab (Los Angeles, California U.S.A) where they were stored in a -70°C freezer until extraction. Urine pH was not adjusted prior to freezing.

Freeze-dried urine samples were derivatized with a benzyltolytriazine reagent to produce benzyl derivatives of alkylphosphate metabolites. A saturated salt solution was added to the tubes and the benzyl derivatives were extracted with cyclohexane and analyzed by gas chromatography with flame photometric detection. Likewise the quality control was made in-house by spiking normal urine sample. Two levels of in-house made urine controls were run. Six dialkylphosphates (DAP) metabolites were measured in the urine samples. The assay was run with a reagent water blank and urine blank. The recovery rate ranged from 80 to 120% of expected value.

The metabolites included in this study were DMP (Dimethylphosphate), DMTP (Dimethylthiophosphate), DMDTP (Dimethyldithiophosphate), DEP (Diethylphosphate), DETP (Diethylthiophosphate), and DEDTP (Diethyldithiophosphate). The limit of detection was 5 ug/l for DMP, DEP, DETP and DMTP, and 10 ug/l for DEDTP and DMDTP. Creatinine was also measured in the urine samples by a colorimetric method (Creatinine Procedure No 555; Sigma Diagnostics, St Louis, Mo). Its measurement was used to adjust results of OP metabolites (ug/gram creatinine) to avoid the variable dilution caused by the different hydration states of the sample donor.

### Data analysis

Data recorded in the questionnaires were introduced in a database Excel. Statistical analysis was performed using the statistical package STATA (version 8.0) for personal computer (Stata Corporation, 702 University Drive East, College Station, TX, USA). Descriptive data were presented as arithmetic means or geometric means and standard deviation (SD), as well as frequencies. The percentage of subjects with detected OPs metabolites in urine (percentage of samples above detection limit for each analyte) was also calculated.

Subjects were also divided in a group with at least one kind of protection against OPs contamination and a group not using protection during pesticides application. In other case, subjects were grouped according the use of OPs pesticides: use frequently (group 1) or less frequently (group 2).

The samples below the respective limit of detection (LOD) were assigned to have concentrations equal to one-half the LOD for statistical analyses [14]. Comparisons between groups were performed with Student's t test (parametric statistics) or Mann-Whitney test (non parametric statistics). A P value below 0.05 was considered as statistically significant.

## Results

The mean age of participants was 34.0 ± 11.5 years (mean ± SD). 54.5% of pesticide applicators had ages between 20–34 years. The period of time that subjects worked as pesticide applicators was 8.55 ± 7.45 years (Table 1). 60.6% of applicators had finished high school.

**Table 1:** Characteristics of selected applicators

**Characteristics**	**No (%)**
***Age (years)***	
20–24	9 (27.3)
25–34	9 (27.3)
35–49	12 (36.3)
>50	3 (9.1)
***No. of years working as applicator***	
2 to 5	16 (48.5)
6 to 9	4 (12.1)
10 to 20	10 (30.3)
>20	3 (9.1)
***Protective measure in use***	
Boots	8 (24)
Apron	1 (3)
Mask	2 (3)
Gloves	0 (0)
Glasses	2 (6)
Respirator	2 (6)
Plastic for the back	15 (46)
Waterproof garment	1 (3)

In relation to protective measures used during pesticide application, 21 out of 33 applicators (64%) reported the use of some kind of protection at work. None of the applicators in Majes Valley used all the protective measures that normally are required. Forty-six percent of them reported the use of only a plastic cover for their back as a measure of protection (Table 1). Nobody used gloves. In addition, 21% of pesticides applicators ate their food within or near to the place of work and 91% used irrigation water for washing their hands before eating food (data not shown).

Ten applicators (30%) reported that they have some kind of knowledge for pesticide handling. 27 out of 33 interviewed subjects (82%) did not ask for information about protective measures when they acquired pesticides in the agro-veterinarian stores (data not shown).

Table 2 shows that most used OPs pesticides were Methamidophos (42%), Triclorphon (42%), Methyl Parathion (30%), Monocrotophos (24%), and Fenitrothion (12%). The less used OPs were Profenophos (9%), Dicrotophos (9%), Pyrazophos (9%), Diazinon (6%), Azinphos methyl (6%), Disulfoton (6%) and Malathion (1%). Fourteen applicators used most frequently methamidophos, ten used frequently Parathion methyl and 8 used Monocrotophos. These three pesticides are considered highly toxic [15].

**Table 2:** Types of organophosphate pesticides most frequently reported to be used by the selected applicators.

**Characteristics**	**No (%)**	**Toxicity**	**Methylated**	**Ethylated**
*Pyrazophos*	3 (9)	1		+
*Coumaphos*	3 (9)	1		+
*Fenitrothion*	4 (12)	1	+	
*Diazinon*	2 (6)	1		+
*Dicrotophos*	3 (9)	1	+	
*Profenophos*	3 (9)	1		+
*Disulfoton*	2 (6)	1		+
*Azinphos methyl*	2 (6)	1	+	
*Malathion*	1 (3)	2	+	
*Triclorphon*	14 (42)	2	+	
*Monocrotophos*	8 (24)	4	+	
*Methyl Parathion*	10 (30)	4	+	
*Methamidophos*	14 (42)	4	+	

1: Low toxicity; 2: Moderate toxicity, 3: High Toxicity; 4: Very High toxicity. + Metabolite present in urine

Moreover, 20 applicators (61%) wore work clothing at home and washed them after getting home, whereas 5 (15%) of the applicators kept work clothing at home and then used them again. Eighteen (55%) kept pesticides in a separate room and 12 (36%) used them as soon as they were bought, while3 (9%) kept them at home. Twenty-six (79%) of the applicators prepared themselves the backpacks ("mochilas") containing the pesticides (data not shown).

Sixty-four percent of the applicators used at least 1 safety measure to avoid pesticide contamination. However, 36% did not use any safety clothing, and 58% of applicators did not use adequate safety devices, mainly due to low economic resources (Table 3). Among the six urine dialkylphosphate metabolites measured, DMP was detected in 72.72% and DEP in 48.48% of applicators. DMDTP was the less frequent metabolite observed (one subject) with a value of 83 ug/g cr. The geometric mean (GM) and geometric mean standard deviation (GSD) of DMP and DEP was 5.73 ug/g cr, (GSD 2.51), and 6.08 ug/g cr. (GSD 3.63) respectively. These results are shown in Table 4.

**Table 3:** Activities of pesticide applicators during the previous week of the study.

**Previous week**	**No (%)**
**Takes shower after a working day**	22 (67)
**How many security components have you used during application the previous week ?**	
None	12 (36)
1 to 2 components	21 (64)
**Reason for not using all protective measures**	
Lack of economic resources	19 (58)
Ignorance	6 (18)
Uncomfortable use	8 (24)
**Have you ever mixed pesticides when fumigating?**	28 (85)
**After pesticide application**	
Were some parts of your body (arms, legs) moist with pesticide?	16 (48)
Were your whole body moist with pesticide?	15 (46)
Were not your body moist with pesticide?	2 (6)

**Table 4:** Concentration of Dialkylphosphates (μg/g creatinine) in the urine of 33 applicators of Majes (Arequipa-Peru)

**Metabolite**	**n**	**% Positv.**	**Mean ± SD**	**GM (GSD)**	**25th Percentile**	**Median**	**75th Percentile**	**90th Percentile**	**Range**
DMP	24	72.72	8.38 ± 7.76	5.73 (2.51)	2.65	6.83	10.02	19.90	1.18–36.67
DEP	16	48.48	14.16 ± 22.21	6.08 (3.63)	1.94	4.36	15.54	37.70	1.01–109.6
DETP	12	36.36	16.07 ± 28.47	5.81 (4.07)	1.65	3.33	16.2	47.46	1.01–147.8
DEDTP	5	15.15	8.09 ± 8.51	5.74 (2.14)	3.19	4.7	10.4	21.06	2.02–38.82
DMTP	5	15.15	4.50 ± 4.20	3.15 (2.29)	1.65	2.38	6.73	11.79	1.01–15.63
DMDTP	1	3.03	8.65 ± 14.74	5.25 (2.24)	3.08	4.39	6.58	20.17	2.02–82.93

n: Number of subjects with determined dialkylphosphate metabolite.

**LOD **(Limit of detection) for: DMP, DMTP, DEP, DETP 5 ug/l. DMDTP, DEDTP 10 ug/l.

• For concentration below the LOD, there was including a value half the detection limit for nondetectable analytes.

In the multivariate analysis we were unable to find an association between 4 parameters of safety practices with urine metabolites of OPs: 1) Training in the proper use of pesticides, 2) use of plastic covers as protective gear, 3) Use of one of the pesticide most frequently used (Methamidophos); 4) Taking a shower at the end of the day of work (Data not shown).

When applicators were grouped according the use of at least one measure of protection or not, the levels of dialkylphosphate metabolites in urine were not different between groups (P > 0.05) (Table 5). Applicators were also grouped as highly frequent users of OPs pesticides (55%) or less frequent user of OPs pesticides (45%), but this was not associated with metabolite level (P > 0.05) (Table 6).

**Table 5:** Dialkyl phosphate (DAP) metabolites in urine in pesticide applicators in Majes Valley, Arequipa, Peru according to use of protective measures.

DAP Metabolites	N	USE OF PROTECTIVE MEASURES		P
		YES	NOT	P

DMP	24	6.73 ± 0.66 (n = 15)	7.05 ± 0.84 (n = 9)	>0.05
DEP	16	20.69 ± 7.97 (n = 12)	6.28 ± 2.34 (n = 4)	>0.05
DETP	12	21.61 ± 10.16 (n = 9)	8.86 ± 3.98 (n = 3)	>0.05
DEDTP	5	6.86 ± 1.07 (n = 3)	6.87 ± 1.27 (n = 2)	>0.05
DMTP	5	4.91 ± 1.35 (n = 3)	4.05 ± 1.06 (n = 2)	>0.05
DMDTP	1	10.69 (n = 1)		

Data are mean ± SE N: number total of subjects for each DAP metabolite. n = number of subjects in each sub-group. P: Probability. NS: Not significant

**Table 6:** Dialkyl phosphate (DAP) metabolites in urine in pesticide applicators in Majes Valley, Arequipa, Peru according to how frequently use OPs pesticides.

Metabolites	N	ORGANOPHOSPHOROUS PESTICIDES USE		
		Frequent Use	Less frequent use	P

DMP	24	6.84 ± 0.67 (n = 13)	6.85 ± 0.81 (n = 11)	>0.05
DEP	16	8.01 ± 1.91 (n = 9)	24.38 ± 11.05 (n = 7)	>0.05
DETP	12	8.16 ± 2.28(n = 5)	27.55 ± 14.15(n = 7)	>0.05
DEDTP	5	5.46 (n = 1)	8.55 ± 1.63 (n = 4)	>0.05
DMTP	5	2.94 (n = 1)	6.59 ± 1.89 (n = 4)	>0.05
DMDTP	1		12.96 (n = 1)	

Data are mean ± SE N: number total of subjects for each DAP metabolite. n = number of subjects in each sub-group. P: Probability. Data were assessed by ANOVA

## Discussion

We studied a population of pesticide applicators in the rural region of the Majes Valley in the Southern Peru. Methamidophos and Trichlorfon were the OPs most frequently used (42%). Both are methylated pesticides. These OPs are considered by the World Health Organization [16] as highly hazardous (Class I-b) and moderately hazardous (Class II), respectively. By comparison, for instance, in the Yakima Valley (Washington State) in the United States the most commonly used pesticide was the azinphos-methyl, classified as level I toxicity [17].

We have measured six dialkylphosphate metabolites in urine of applicators workers from the Majes Valley, and shown that 76% of them showed at least one OP metabolite in urine. Sanchez-Peña et al. [18] in Mexico found that 87% of agricultural workers have at least one OP metabolite in urine.

In the Majes Valley, the most common metabolite found was DMP (72.72%) followed by DEP (48.48%). Other studies with measurements of dialkylphosphates showed that DMP was also the most common metabolite in urine [19,20].

Some studies in US farm workers showed that DMP was the most frequently detected metabolite (33%) followed from DMTP detected in 28% of the workers [21]. However, others authors in Washington, US showed that DMTP was more frequent than DMDTP and DMP [17]. DMTP was found in-subjects without known exposure to OPs [4] and it has been suggested that DMTP and DETP excretion may not be specific to pesticide exposure or that other phosphorylated compounds may interfere with the analysis [22].

Sanchez-Peña et al., [18] in farm workers from Mexico found that Diethylthiophosphate (DETP) was the most frequent OP metabolite in urine samples, indicating that compounds derived from thiophosphoric acid were mainly used. In that study, diazinon was frequently used. Diazinon is an ethylated OP and therefore it is logical that ethylated OPs metabolites will be present in urine of these workers. In Majes Valley, the methylated OPs were most frequently used (i.e Methamidophos). Therefore it is not surprising that we found that methylated OP metabolites in urine were more frequently observed. One other study in El Salvador has shown that the use of Methamidophos leads to methylated OP excretion [4]. Moreover, dimethyl phosphate (DMP) is a metabolite of phosphamidon, mevinphos, dicrotophos, monocrotophos, dichlorvos, and trichlorfon [23] and several of these OPs pesticides were used in Majes Valley.

In other studies, the frequencies of detection of OPs metabolites found in urine of farm workers were as follows: 96 and 94% [20]; 83 and 99% CDC [21]; 51% and 68% [18] and 53 and 71% (NHANHES 1999–2000) for DMP and DEP, respectively. In Majes Valley, the frequencies of detection of DMP and DEP were 72.72% and 48.48% respectively. The data of OPs metabolites in urine should be interpreted carefully since exposure to these metabolites may also occur from dietary and or other environmental sources [24].

Geometric mean for DMP and DEP levels found in the pesticide applicators of the Majes Valley in Peru was 6 times higher to those found in USA [10] in non occupationally exposed men aged 20–59 years suggesting that values were related to direct pesticide exposure rather than exposure from another sources. This suggests that pesticide applicators in Majes Valley have a high risk of exposure and that high levels may be due to inappropriate practice of safety measurements of the guidelines for OPs handling.

We surprisingly found that 36% of the applicators did not use any kind of protection. According to the interviewers, the main reason for not using protective clothing during pesticide application was economic. The same was found by other authors in agricultural farm workers in the Gaza Strip, Palestine [25]. The second reason for non-use was that they are not aware of Protection Guidelines. These Guidelines suggest the use of protective: work clothing, including protective gloves, footwear, outer garments, and eye and face protection,. In fact, 46% of the applicators used a plastic cover to protect their backs as the only measure of protection against exposure to pesticides. These measures are usually used independently of the type of the pesticide. Our results showed no differences in OPs metabolites levels between applicators using or not using any kind of protective measures, suggesting that safety practices used by applicators in Majes Valley are inadequate.

Applicators were also grouped according as if they are high frequently users or low frequently users of OPs pesticides showing no differences in the values of OPs metabolites in urine, suggesting that measurements were related to the last pesticide application, one day before the urine sample was requested. Our findings suggest the need for implementation of appropriate clothing and equipment for protection as well as a continuous training in the use of pesticides by the formulators, applicators, and farmers from this region. This concern should be extended to the farmers families since non-occupational exposure to agricultural pesticide can also be an important cause of contamination. For example, exposed farmers have been shown to track in residues, and keep contaminates containers near the house [4].

The different kind of protective equipments also influence the exposure to pesticides. In the present study 100% of the applicators do not use gloves for protection and 93.9% do not use masks for protection. Alavanja et al., [26] observed that 76% and 47% of farmers from Iowa (USA) used chemical-resistant gloves and masks, respectively. However, in North Carolina the prevalence of protective gear (resistant gloves and masks) was lower (39.4% and 33.2%, respectively).

Our study showed that pesticide applicators get information but not training about handling OPs from the dealers. Dealers are not adequate persons to train farm-workers about the handling pesticides as it has been seen previously [15].

Another problem is the storage of OPs pesticides after they are acquired by applicators. Yassin et al. [25] in the Gaza Strip, Palestine found that 78% of farmworkers stored pesticide containers on the farm, whereas 18% stored them at home. In Majes Valley, the 55% of interviewed pesticide applicators reported that they use a separate room to keep the OPs. The rest of workers maintained the OPs at home. This is very dangerous behavior since massive contamination may be a consequence. The pesticide poisoning deaths of 24 children in an isolated Peruvian village (Tauccamarca) make a compelling case that corporate accountability for pesticide poisonings in developing countries should be examined from a human rights perspective [27].

## Summary

Our report, the first assessed for Peru, aimed to determine the concentration of dialkylphosphate metabolites in urine of pesticide applicators and the frequency of pesticide applicators with OPs metabolites in urine. The study showed that 76% of applicators had at least one metabolite detected in urine samples suggesting inadequate measures for protection. Another report in Mexico showed also that 87% of the study workers had at least one OP metabolite in their urine at the time of the study [18] suggesting that contamination with OPs pesticides is a problem in Latin American farmers. The majority of applicators interviewed were not aware that the use of protective clothing can prevent the detrimental effects of pesticides. It is crucial that people get information about the risks of the use of pesticides in an inadequate way. This reinforces the idea that these compounds are too much toxic for people who use them in hot climates live close to their work sites with limited access to protective equipment, and no practical means for using and wearing adequate equipment. It is important to consider preventive options like elimination or substitution of certain compounds, reduction in use, integrated pest management, organic methods, among others.

## List of Abbreviations

OPs: Organophosphorus Pesticides

DAP: Dialkyl phosphate Pesticides

DMP: Dimethylphosphate

DMTP: Dimethylthiophosphate

DMDTP: Dimethyldithiophosphate

DEP: Diethylphosphate

DETP: Diethylthiophosphate

DEDTP: Diethyldithiophosphate

GC/FDP: Gas chromatography with flame photometric detection method.

GM :Geometric mean

GSD :Geometric mean Standard Deviation

ug/g cr : Microgram per gram of creatinine

## Declaration of competing interests

The author(s) declare that they have no competing interests.

## Authors' contributions

**SY **conceived of the study, participated in its design, coordination, execution, analysis and interpretation of the data and drafted the manuscript giving final approval of the version to be published

**KS **provided comments on the manuscript and has given the final approval of the version to be published

**AC **participated in the analysis and interpretation of the data

**FC **participated in the coordination in the study area.

**GG **have been involved in analysis, interpretation of data, drafting the manuscript and has given the final approval of the version to be published.
